# Labourers' Wages and Labourers' Homes

**Published:** 1888-05-12

**Authors:** Earl Grey


### Labourers' Wages and Labourers' Homes.

By the Right Honourable Earl Grey, K.G., G.C.M.G.

The articles entitled "London's Unsolved Problem" and "London's Problem Solved," in the January number of The Hospital and its Special Supplement, have brought under consideration a subject of great importance and of great difficulty. They point out in a very striking manner the evil and the dangers that arise from there being now brought together in London an enormous population, of which a large proportion in many parishes consists of persons whose life is habitually a wretched one, and who are frequently reduced to such absolute want as to make assistance in some shape or other indispensable, in order to save them from perishing. In the weeks that have gone by since those articles were published there has been much discussion on the question they raise; but though this discussion has brought out much useful information and some valuable suggestions, it seems as yet to have produced little practical advantage beyond that of having demonstrated the extreme difficulty of the subject, and the urgent need there is for dealing with it. Unfortunately, while the existence of great evils has been only too clearly proved, it does not appear that any means by which they could be effectually corrected have hitherto been discovered, for a careful examination of the scheme propounded in the article which is called 'London's Problem Solved" does not, in my opinion, lead to the conclusion that it has a good claim to be that described. The main object of this and a preceding article is to point out means for giving more efficient aid than they can now obtain to two different classes of distressed inhabitants of London, for whom it is intended that something must be done. The first class consists of persons who, from age or infirmity, are unable to maintain themselves in reasonable comfort by their own exertions; the other is composed of that large number of persons who are too often to be found in London, who, though willing and able to work, cannot find regular and sufficiently remunerative employment. With regard to the first of these classes it is remarked in p. viii. of the Special Supplement to the January number of The Hospital that "certain persons must of necessity become paupers, and that many of them will become so through no fault of their own; . . . that it is right and proper they should be relieved;" and therefore it is said to be "desirable that deserving cases shall be relieved with as much regard to their own comfort and convenience as possible." Then follows an argument which, if I understand it rightly, is in favour of some relaxation of the rules under which relief is now administered by the poor law authorities, and especially in the matter of a less strict adherence to the rules of refusing, except in very special circumstances, out-relief to the able-bodied. It then goes on to ask: "Can it be either wise or humane to reckon all paupers together as persons against whom society must always be in an attitude of defence? Are there not—must there not always be—a number of thoroughly well-conducted persons who, as they become older and feebler, will insensibly graduate downwards until the dreadful region of pauperism is reached; and are these then to be pitched into, and for ever more to remain in the dreadful cauldron of half-thieves, half-drunkards, incapable fools, and hopeless reprobates, who constitute a large proportion of the pauper army?" A question might well be raised, whether it is indeed true that "there must always be a number of *thoroughly* well-conducted persons who, as they become older and feebler, inevitably sink into distress?" In a great majority of cases, though there may have been no positive misconduct to account for the distress of those for whom the above appeal is made, it is a greater or less deficiency of energy and self-denial in earlier life which has prevented them from making proper provision for the sure coming of old age and weakness. My own belief is that if the true causes which have brought men to the necessity of seeking for help when their failing strength leaves them without the power of earning their own livelihood could be ascertained, it would be found that there are very few indeed whose difficulties would not be found to be due, in part at least, to their own fault. Without, however, dwelling upon this point, I fully admit that there are a large number of persons to whom it is desirable that assistance of a more generous character should be afforded than that which they can receive under a strictly administered poor law; but I hold that such assistance ought not to be granted from the rates. In the first place, a large number of those by whom the rates are paid have no small difficulty in maintaining themselves by their own exertions, and it would be unjust to them to increase the burthen they have to bear by granting more from the rates than is strictly necessary to persons not very much worse off than themselves. And further, what is proposed would involve a return to some of the abuses and evils which the Poor Law Amendment Act of 1834 corrected. Among these evils there were perhaps hardly any so bad as those which had arisen from the practice of giving relief in aid of wages. The admirable report of the Commissioners of Inquiry on which the Act of 1834 was founded, demonstrated that this practice had contributed far more than any other cause to bring down the agricultural population in the south of England to the terrible condition of distress to which they had been then reduced. To adopt the suggestion that is made "to relax the stringency [of the present rules] in the case of the aged or those disabled by illness, to such an extent at any rate as to allow them to maintain themselves in part by their own exertions," would in reality be to return to the practice so decisively condemned by the Bishop of London (Blomfield) and his colleagues in the Commission of Inquiry, and would be sure to produce similar effects. The competition of persons who received part of what was necessary for their support from the rates, would necessarily tend to bring down the wages of independent labourers in the trades in which these assisted persons might find employment. The other evil consequences which have invariably been found to result from any system of public relief for the poor, unless very strictly administered, would follow from any relaxation in the existing system beyond what is already provided for by the law; more especially it would tend to discourage habits of self-denial and providence for the future among the working classes. But what cannot safely be done in the way of giving a greater amount of relief from the rates to persons in distress who come under the description of "deserving cases," may be most properly and usefully accomplished by private benevolence. The money now given in charity, which is too often ill-directed, would probably be sufficient to afford as much assistance to the distressed as is really needed or could be granted with advantage, and if more money can be shown to be wanted there is no doubt it would be cheerfully given; but what is much more required than additional supplies of more money is a more complete system for exercising a judicious use of that which is already available. To this object the efforts of the Charity Organisation Society have been long and usefully directed, and the articles in The Hospital give an interesting account of the organisation which has been created in Paddington for this purpose, adding the excellent suggestion that a similar organisation should be adopted in the other Parliamentary districts of the metropolis. "Deserving cases" might thus be treated with greater liberality than would be safe in granting relief from the rates. Experience has demonstrated the danger, or rather the certainty of abuses, arising if the plain rule is departed from, that the claim of the destitute upon funds levied from the public by law is limited by their actual wants. The evils produced by a lax administration of relief under the poor law are not to be feared from generous assistance afforded to those who are proper objects of compassion by judicious and well-regulated private benevolence. There is, however, no occasion for dwelling on these considerations, which have been so often insisted upon, and are so generally accepted. Though they appear to have been for a moment lost sight of in the sentences I have adverted to, they must be familiar to the able writer of the articles from which I have quoted them, and his useful remarks upon the importance of having a good organisation to secure the judicious application of the large amount of money freely given for the relief of distress by the charity of individuals, implies that he looks to this source rather than to the poor rate for the means of affording the generous aid he properly desires to obtain for those he describes as "deserving objects."

Little difficulty need, therefore, be anticipated in deciding on the means to be adopted for the relief of that class of persons who are in distress from their inability to earn their own subsistence, but to whom it is desirable to give more assistance than they now receive from the poor rates. What ought to be done for the other and larger class of the distressed inhabitants of London, consisting of those who are willing and able to work, but who cannot find sufficient employment, is a much more embarrassing question. I have already recognised the truth of what has been asserted as to the evil and the danger resulting from having a large number of the dwellers in London living habitually on the brink of destitution, and as to its being most desirable to find a remedy for so great an evil; but I cannot see the faintest ground for hoping that a real and permanent remedy for it would be provided by any of the various measures that have been proposed for that purpose. I am myself totally unable to suggest other measures more likely than those already recommended to prove successful in effecting such an improvement in the present state of things as is most earnestly to be desired, and I must venture to express my strong doubt whether it is possible that much change for the better should be speedily accomplished by any means that could be adopted.

*(To be continued.)*

